# An *IGF-I promoter *polymorphism modifies the relationships between birth weight and risk factors for cardiovascular disease and diabetes at age 36

**DOI:** 10.1186/1472-6823-5-5

**Published:** 2005-06-01

**Authors:** Saskia J te Velde, Elisabeth FC van Rossum, Paul G Voorhoeve, Jos WR Twisk, Henriette A Delemarre van de Waal, Coen DA Stehouwer, Willem van Mechelen, Steven WJ Lamberts, Han CG Kemper

**Affiliations:** 1 Institute for research in extramural medicine (EMGO), VU University Medical Center, Amsterdam, The Netherlands; 2 Department of Internal Medicine, Erasmus MC, Rotterdam, The Netherlands; 3 Department of Pediatric Endocrinology, VU University Medical Center, Amsterdam, The Netherlands; 4 Department of Clinical Epidemiology and Biostatistics, VU University Medical Center, Amsterdam, The Netherlands; 5 Institute for Cardiovascular Research and Department of Internal Medicine, VU University Medical Center, Amsterdam, and Department of Medicine, University Hospital Maastricht, Maastricht, The Netherlands; 6 Department of Social Medicine and Body@Work research centre for physical activity, work and health TNO-VU, VU University Medical Center, Amsterdam, The Netherlands

## Abstract

**Objective:**

To investigate whether *IGF-I promoter *polymorphism was associated with birth weight and risk factors for cardiovascular disease (CVD) and type 2 diabetes (T2DM), and whether the birth weight – risk factor relationship was the same for each genotype.

**Design and participants:**

264 subjects (mean age 36 years) had data available on birth weight, IGF-I promoter polymorphism genotype, CVD and T2DM risk factors. Student's t-test and regression analyses were applied to analyse differences in birth weight and differences in the birth weight – risk factors relationship between the genotypes.

**Results:**

Male variant carriers (VCs) of the *IGF-I promoter *polymorphism had a 0.2 kg lower birth weight than men with the wild type allele (p = 0.009). Of the risk factors for CVD and T2DM, solely LDL concentration was associated with the genotype for the polymorphism. Most birth weight – risk factor relationships were stronger in the VC subjects; among others the birth weight – systolic blood pressure relationship: 1 kg lower birth weight was related to an 8.0 mmHg higher systolic blood pressure

**Conclusion:**

The polymorphism in the promoter region of the *IGF-I gene *is related to birth weight in men only, and to LDL concentration only. Furthermore, the genotype for this polymorphism modified the relationships between birth weight and the risk factors, especially for systolic and diastolic blood pressure.

## Background

Insulin-like growth factor-1 (IGF-I) is a peptide that is involved in fetal growth and cell differentiation [1,2] In addition, it has been suggested that this peptide plays a role in the regulation of glucose homeostasis and cardiovascular function [3-6] (Lower IGF-I levels are also associated with increased levels of serum low-density lipoprotein (LDL) [7]. IGF-I also plays a role in plaque development [8].

A genetic polymorphism comprising a variable length cytosine-adenine (CA) repeat sequence in the promoter region of the *IGF-I gene *has been identified, and is thought to influence the transcription rate of IGF-I, which in turn affects serum IGF-I levels [9]. Since IGF-levels are associated with fetal growth and adult risks for cardiovascular disease (CVD) and type 2 diabetes (T2DM), it has been suggested that the polymorphism in the promoter region of the *IGF-I gene *might be relevant to the fetal origins hypothesis. This hypothesis suggests that an adverse environment during the intra-uterine period negatively affects fetal growth (often estimated by birth weight), and results in adaptations that permanently change the structure and functions of the body, which leads to an increased risk for disease, such as CVD and T2DM, at adult age [10]. An alternative hypothesis is that impaired fetal growth and increased risk for CVD and T2DM share a common genetic factor [11,12] A study performed in Rotterdam in the Netherlands has recently shown that the absence of the wild type allele (192 base pair (bp)) in the promoter region *IGF-I gene *was related to lower birth weight [13]. In addition, the intra-uterine environment may interact with genetic polymorphisms [14]. This has already been found in other studies, two of which concerned on birth weight and genetic factors in insulin metabolism [15-17] These data raise the issue of whether or not birth weight also interacts with the *IGF-I gene*.

In the Amsterdam Growth and Health Longitudinal Study (AGAHLS), data on birth weight, and risk factors for CVD and T2DM have been collected. The *IGF-I gene *has now been analysed for the 192 bp polymorphism [9,13], in order to address the following four research questions: 1) Is the *IGF-I promoter *polymorphism genotype associated with birth weight? 2) Is *IGF-I promoter *polymorphism genotype associated with risk factors for CVD and T2DM? 3) Is birth weight associated with risk factors for CVD and T2DM and finally 4) Do intra-uterine environment and the *IGF-I promoter *polymorphism genotype interact? Or, in other words, is the association between birth weight and risk factors for CVD and T2DM different for each genotype of this polymorphism?

## Methods

### Participants

The Dutch population described in this study are all participants of the AGAHLS. The AGAHLS is an observational study with repeated measurements, which started in 1976 to monitor boys and girls with a mean age of 13 years [18]. During the most recent measurement in which 433 subjects participated, subjects had than reached a mean age of 36 years, information was obtained concerning birth weight, *IGF-I promoter *polymorphism genotype, glycated hemoglobin (HbA_1c_), carotid intima-media thickness (IMT), blood pressure, anthropometry and serum low density lipoprotein (LDL) cholesterol levels. Three-hundred-ninety-one subjects completed the birth-weight questionnaire of whom 380 had written information or information from parents. For the purpose of the present study, subjects who were born pre-term (before 37 weeks of gestation, 27 subjects), were one of a twin (11 subjects) or were of non-Caucasian ethnicity (9 subjects) were excluded. Another 69 subjects were not taken into account for the analyses because of errors or missing values in the genotyping or risk factor measurements. Finally, for 264 subjects (152 women) there were complete data sets on the *IGF-I promoter *polymorphism genotype, birth weight, and adult risk factors for CVD and T2DM. Subjects included for the analyses were smaller (1.76 m vs. 1.77 m, p = 0.030) and had less fat-free mass (56.5 kg vs. 57.9 kg, p = 0.056) compared to subjects not included for these analyses, but with data on the outcomes available (N = 109). On all other outcome variables the groups did not differ. (Some outcome measures had missing values, which were due to error in the specific measurement.) All subjects were apparently healthy at the time of the measurements, and none had been diagnosed with T2DM. All subjects gave written informed consent and the Medical Ethical Committee of the VU University Medical Center approved the protocol.

### Birth weight

Data on birth weight were obtained by means of a questionnaire. The questions concerned birth weight, gestational age, being one of a twin, and ethnicity, and subjects were also asked about the source of the information. Only those who had received the information from their parents or had it in written documents were included, as this has been shown to be a valid method [19,20] Subjects born preterm (gestational age < 37 weeks) were excluded, this may have independent effects on adult health or influence the relationship between birth weight and adult health [21-23]. Twins were excluded because they have different fetal growth patterns, which might cause error when analysing the relationship between birth weight and adult health outcomes. Subjects who retrieved the birth weight information from their parents' memory (n = 112) had slightly higher mean birth weights (3.54 ± 0.53 kg) compared with the subjects who retrieved the requested information from written documents (n = 152; 3.44 ± 0.48 kg), but this difference was not significant (p = 0.10). Furthermore, they did not differ significantly on any of the outcome measures.

### Polymorphism in the promoter region of the IGF-I gene

*IGF-I promoter *polymorphism genotypes were determined as described earlier [9]. In brief, DNA was isolated using standard methods. PCR was performed in a final volume of 10 μL containing 10 ng DNA, 10* Gold (Au) buffer (Perkins and Elmer), 200 M dNTP, 30 pmol of each primer, 3 mM MgCl2, 0.5 U Ampli Tag Gold polymerase (Perkins and Elmer). The PCR program concisted of 30 cycli of 30 sec 95°C, 30 sec 55°C and 30 sec 72°C and additionally 5 min of denaturation at 95°C before the first cycle and an extension of 10 min. at 72°C after the last cycle. Forward primers were labelled with FAM (Weber & May 1989) to determine the size of the PCR products by fragment analysis (ABI-Prism genetic analyser with Genescan 2.1 software). The Genescan 350/500 Tamra was used as internal size standard within the fragment analysis.

Rietveld et al[24] recently demonstrated that subjects who were homozygous for the 192 bp or the 194 bp allele had comparable IGF-I blood levels, while individuals who were homozygous for either alleles shorter than 192 bp or longer than 194 bp had significantly lower serum IGF-I levels. Therefore, we decided to regard all subjects who were homozygous for 192 bp or 194 bp, or were carrier of a 192 bp allele and a 194 bp allele as wild types (WTs). Consequently, all subjects who were carrier of a variant allele, which is either shorter than 192 bp or longer than 194 bp, were grouped as variant carriers (VCs).

### Risk factors for CVD and T2DM

The following risk factors for CVD and T2DM were measured: body mass index (BMI), waist circumference, waist-to-hip ratio (WHR), total fat mass (FM), total fat-free mass (FFM), carotid intima-media thickness (IMT), systolic and diastolic blood pressure (SBP, DBP), resting heart rate, LDL cholesterol levels and HbA_1c _as an estimate of glucose metabolism (unfortunately no glucose measures were available). BMI was calculated as body weight (kg) divided by body height (m^2^). Standing height was measured with a stadiometer to the nearest 0.001 m. Body weight (kg) was measured to the nearest 0.1 kg using a spring balance scale (Van Vucht, Amsterdam, The Netherlands), with subjects dressed only in underwear. Waist (at the level of the umbilicus) and hip circumference were measured with a flexible steel tape to the nearest 0.1 cm. WHR was calculated as the ratio between waist circumference and hip circumference. Fat mass (FM) was estimated from four skinfolds (biceps, triceps, subscapular and supra iliacal) with the Durnin and Womersley equation [25]. The four skinfolds were measured according to standard procedures [26]. FFM was calculated by subtracting FM from body weight.

IMT of the right common carotid artery was obtained by an ultrasound scanner equipped with a 7.5 MHz linear array probe (Pie Medical, Maastricht, The Netherlands), as described elsewhere in more detail [27-29] SBP and DBP were assessed in the left arm at 5-minute intervals with an oscillometric device (Colin Press-Mate, model BP 8800, Komaki-City, Japan) during the entire period of ultrasound imaging when the subjects were lying in a supine position. The mean value over this entire period was calculated. Resting heart rate was measured with the same device as used for the blood pressure measurement. The mean value over this measurement period was calculated and used in the analyses.

Serum LDL and HbA_1c _(%) were measured from blood samples (10 ml) taken from the antecubital vein between 8.30 and 12.30 a.m. with subjects in a non-fasting state. Standard methods were used to analyse the LDL concentration and external quality control took place with target samples from a World Health Organisation reference laboratory (Lipid Standardization Laboratory, Atlanta, USA). HbA_1c _was determined by non-exchange high performance liquid chromatography with a modular Diabetes Monitoring System (Bio-Rad, Veenendaal, the Netherlands).

### Data-analyses

A t-test was used to analyse differences in birth weight between the *IGF-I promoter *polymorphism genotypes. Multiple linear regression analyses were applied to study the associations between birth weight and the risk factors for CVD and T2DM. The results of the regression analyses were presented as regression coefficients (β) and the corresponding 95% confidence intervals (CI) for two different models. The first model was a crude analysis, only adjusted for gender (and for SBP and DBP in case of IMT). The second was further adjusted for adult body weight.

To investigate whether the relationship between birth weight and the risk factors for CVD and T2DM were modified by the *IGF-I promoter *polymorphism, multiple linear regression was performed between birth weight and all risk factors (all as continuous variables) for the two genotypes separately.

All analyses were performed with the Statistical Package of Social Science (SPSS, Chicago, USA) version 10.1. The statistical significance was set at p-value ≤ 0.05.

## Results

Table 1 gives allele and genotype frequencies in this cohort. As can be seen, of all 264 subjects, 189 (111 women) were WTs for the *IGF-I promoter *polymorphism. The remaining 75 subjects (41 women), 4 were homozygous for alleles with variant CA repeats and 71 were heterozygous for variant alleles. The distribution of genotypes was in Hardy-Weinberg equilibrium (p = 0.17).

**Table 1:** Allelic and genotype frequencies for the bp repeat polymorphism at the promoter region of the IGF-I gene

Allele (bp length)	Frequency N (%)	Genotype		Frequency N (%)
176	3 (0.5)	WT	192/192	105 (39.8)
188	10 (1.9)		192/194	78 (29.5)
190	23 (4.4)		194/194	6 (2.3)
192	341 (64.6)		total	189 (71.6)
194	108 (20.5)	VC	192/x	53 (20.0)
196	38 (7.2)		194/x	18 (6.8)
198	5 (1.0)		x/x	4 (1.5)
			total	75 (28.4)

X, alleles with bp lengths other than 192 or 194; WT, Wild Type; VC, Variant Carrier

Population characteristics on all measured variables are presented in Table 2, stratified according to gender and genotype. In men, the mean birth weight was 0.2 kg lower in the VC group than in the WT group (p = 0.009). In women, no significant differences in birth weights between the genotypes were observed (p = 0.755).

**Table 2:** Characteristics of the adult population of the Amsterdam Growth and Health Longitudinal Study

	Men		Women	
	WT	VC	WT	VC
	Mean ± SD	Mean ± SD	Mean ± SD	Mean ± SD
Birth weight (kg)	3.64 ± 0.47	3.40 ± 0.39	3.44 ± 0.53	3.41 ± 0.52
Height (m)	1.84 ± 0.08	1.83 ± 0.07	1.71 ± 0.06	1.72 ± 0.06
Weight (kg)	84.1 ± 10.8	85.2 ± 10.8	67.5 ± 9.3	69.2 ± 11.8
Body mass index (kg/m2)	23.5 ± 2.6	24.5 ± 2.4	22.4 ± 2.9	22.8 ± 3.8
Fat-mass (kg)	13.7 ± 5.0	15.3 ± 5.6	17.7 ± 5.3	18.4 ± 6.4
Fat-free mass (kg)	66.1 ± 6.6	66.3 ± 5.7	47.3 ± 4.5	48.7 ± 5.9
Waist circumference (cm)	85.3 ± 7.5	85.9 ± 8.8	73.3 ± 8.9	73.4 ± 8.0
Waist-to-hip ratio	0.95 ± 0.04	0.96 ± 0.05	0.83 ± 0.08	0.82 ± 0.08
Systolic blood pressure (mmHg)	121.0 ± 8.9	122.7 ± 14.4	110.7 ± 10.6	111.8 ± 12.0
Diastolic blood pressure (mmHg)	66.4 ± 6.4	67.2 ± 8.6	63.1 ± 7.2	61.6 ± 6.9
LDL concentration (mmol/l)	3.22 ± 0.80	3.52 ± 0.87	2.80 ± 0.81	2.97 ± 0.70
Carotid intima-media thickness (mm)	0.628 ± 0.099	0.636 ± 0.095	0.616 ± 0.086	0.631 ± 0.101
Resting heart rate (b/min)	73 ± 12	69 ± 11	72 ± 12	70 ± 12
Glycated hemoglobin (%)	5.3 ± 0.5	5.3 ± 0.3	5.3 ± 0.4	5.3 ± 0.3

Data is presented as means ± standard deviations (SD),

†WT, Wild type; VC, Variant carrier LDL – low lipoprotein

Subjects in the VC group had significantly higher LDL concentrations (p = 0.039). No other significant differences between the genotypes were observed.

Table 3 presents the results of the linear regression analyses for the relationship between birth weight and risk factors for CVD and T2DM. It was found that 1 kg higher birth weight was associated with 2.55 kg more FFM. However, this association decreased and lost significance after adjustment for adult body weight. In addition, birth weight was found to be associated with SBP in such a way that 1 kg lower birth weight was related to a 3.05 mmHg higher SBP. No other significant associations were observed between birth weight and risk factors for CVD and T2DM.

**Table 3:** Results of the linear regression analyses for the relationship between birth weight and adult risk factors for CVD and DM-2

	Crude†		Adjusted ‡	
Outcome	β	95%CI	β	95%CI

Body mass index (kg/m^2^)	0.445	[-0.264; 1.154]		
Fat-mass (kg)	0.962	[-0.361; 2.285]	-0.663	[-1.605; 0.208]
Fat-free mass (kg)	2.553***	[1.224; 3.882]	0.714	[-0.085; 1.513]
Waist circumference (cm)	1.760	[-0.372; 3.892]	-1.013	[-2.253; 0.227]
Waist-to-hip ratio	0.012	[-0.005; 0.028]	0.006	[-0.011; 0.022]
Systolic blood pressure (mmHg)	-1.387	[-4.082; 1.309]	-3.050*	[-5.626; -0.475]
Diastolic blood pressure (mmHg)	-0.692	[-2.445; 1.071]	-1.608	[-3.329; 0.113]
Resting heart rate (b/min)	1.941	[-0.932; 4.815]	1.861	[-1.081; 4.803]
Low density lipoprotein (mmol/l)	-0.079	[-0.277; 0.118]	-0.154	[-0.351; 0.044]
Carotid intima-media thickness (mm) ∥	0.011	[-0.012; 0.034]	0.011	[-0.012; 0.035]
Glycated hemoglobin (%)	-0.013	[-0.109; 0.082]	-0.025	[-0.123; 0.072]

Data is presented as regression coefficients and corresponding 95% confidence intervals (CI)

CVD – cardiovascular disease; DM-2 – type 2 diabetes mellitus

† Crude, only adjusted for gender; ‡, Adjusted, further adjusted for adult body weight

* p < 0.05 *** p < 0.001

∥ also adjusted for systolic and diastolic blood pressure

Table 4 shows the results of the linear regression analyses between birth weight and the risk factors for CVD and T2DM, stratified according to *IGF-I promoter *polymorphism genotypes. In most of the associations studied, the regression coefficient for birth weight was highest in the VC group, indicating a stronger effect of birth weight on the outcome variable. This difference was most marked in the association with SBP, in which a 1 kg lower birth weight was related to an 8.0 mmHg increase in adult SBP in the VC group, compared to a 1.4 mmHg increase in the WT group. Although, the difference between the VC group and WT group was not statistically significant (p = 0.08) for these kind of 'interactions' normally a higher significance level is used. In addition, the relationships between birth weight and DBP and FM were significant in the VC group and not in the WT group (p = 0.06 for the difference between VCs and WTs for DBP). The differences between VCs and WTs regarding all other relationships showed p-values > 0.10.

**Table 4:** Analyses stratified according to genotype for the relationship between birth weight and risk factors for CVD and DM-2

	Wild type		Variant carrier	
Risk factor	β	95% CI	β	95% CI

BMI (kg/m^2^)	0.475	[-0.312; 1.262]	0.777	[-0.835; 2.338]
Fat mass (kg)	-0.217	[-1.337; 0.904]	-1.929*	[-3.742; -0.116]
Fat free mass (kg)	0.780	[-0.212; 1.722]	0.473	[-0.897; 1.844]
Waist circumference (cm)	-1.380	[-2.822; 0.061]	0.040	[-2.588; 2.668]
Waist-hip ratio	0.002	[-0.016; 0.021]	0.021	[-0.015; 0.057]
Systolic blood pressure (mmHg)	-1.497	[-4.265; 1.272]	-8.038*	[0.014; -14.391]
Diastolic blood pressure (mmHg)	-0.599	[-2.553; 1.355]	-5.073**	[-8.845; -1.301]
LDL cholesterol (mmol/l)	-0.138	[-0.369; 0.093]	-0.076	[-0.491; 0.339]
Resting heart rate (b/min)	2.540	[-0.865; 5.946]	-1.995	[-8.277; 4.286]
Carotid intima-media thickness (mm)‡	0.010	[-0.017; 0.036]	0.020	[-0.035; 0.075]
Glycated hemoglobin (%)	-0.019	[-0.138; 0.101]	-0.025	[-0.207; 0.157]

Data is presented as regression coefficients (β) and their corresponding 95% confidence intervals (CI)

CVD – cardiovascular disease; DM-2 – type 2 diabetes mellitus

Models were adjusted for gender and adult body weight * p < 0.05 ** p < 0.01

‡ also adjusted for systolic and diastolic blood pressure

For the associations between birth weight and adult FFM, waist circumference, LDL and resting heart rate, the regression coefficients were highest in the WT group, although none of these associations were statistically significant.

## Discussion

In this study, the associations between a polymorphism in promoter region of the *IGF-I gene*, birth weight, (as a measure of intra-uterine growth), and risk factors for CVD and T2DM were investigated, in order to obtain more insight into the genetic aspects of the fetal origins hypothesis [1,5,6,30,31](The results of the present study demonstrate that men who were carriers of one or two variant allele(s) of the *IGF-I gene *had significantly lower birth weights. However, this trend was not observed in women. It is not clear, why this association was absent in women, as no other study has reported gender differences in the association between *IGF-I *genotype and birth weight [13,32,33]. Therefore, the gender difference observed in this study might be a result of chance

So far, results on the association between *IGF-I *genotype and birth weight have been conflicting. Vaessen et al. reported that absence of the wild type allele (192 bp) resulted in a lower birth weight, but subjects who were heterozygous for the wild type allele did not differ in birth weight from the homozygous subjects [13]. Nevertheless, Vos et al. [33], Frayling et al. [32] and Day et al [34] could not confirm these findings. These conflicting results could be due to differences in the population backgrounds, but also to the way in which subjects were classified per genotype. In the present study, an alternative method was used, in which the allele with 194 bp was also considered as a wild type allele, based on the observations made by Rietveld et al[24]. Subjects previously categorised as VC were now categorised as WT (e.g. subjects with genotype 192 bp/194 bp, or 194 bp/194 bp). Therefore, the VC group in other studies was actually heterogeneous, which may explain discrepant observations. To investigate this possible explanation, we investigated the characteristics of the subjects who would have been categorised as VC according to the traditional classification, and were now categorised as WT (84 subjects). The men in this group had a mean birth weight of 3.63 kg (± 0.51 kg), which is comparable with the men who were categorised as WT in both classification methods (3.64 ± 0.43 kg). The women in this group had a mean birth weight of 3.44 kg (± 0.53 kg), which is exactly the same as the women classified as WT in both methods. Besides this, the three groups were also different with regard to the interaction between birth weight and SBP and DBP in a way that the relationship between birth weight and adult SBP and DBP was strongest and significant in the 'constant' VCs (β = -8.0 for SBP and β = -5.1 for DBP), and weak and not significant in the two other groups. These results suggest that the alternative method used in the present paper discriminates better between the genotypes with regard to the observed health outcomes.

The results concerning risk factors for CVD and T2DM showed an increased risk in the VC group solely for LDL concentrations, all other risk factors did not differ between WT and VC groups. That only one risk factor was significantly different between the groups might be real or a result of chance, since we tested several associations. Moreover, the relationship between *IGF-I genotype *and LDL concentrations disappeared after adjustment for BMI and there were no differences in HDL concentrations (p = 0.255, data not shown). Several studies have shown that lower IGF-I bio-activity is related to higher incidence of atherosclerotic cardiovascular disease, higher carotid IMT values, lower levels of HDL cholesterol, and impaired glucose tolerance [5-7,35].(However, other studies have failed to show these associations [36-38] Until now, observations have thus been inconsistent, which may be due to other factors that affect cardiovascular health, insulin metabolism and serum IGF-I levels, such as nutrition and endocrine factors. One should, however, realise that the subjects of the present study were still rather young (i.e. 36 years), and that a longer exposure to lower IGF-I bioactivity might be necessary to induce unfavourable levels of risk factors for CVD and/or T2DM.

Another aim of the present study was to investigate whether birth weight was associated with risk factors for CVD and T2DM. This was found to be the case for FFM, however this association decreased after adjustment for body weight. When studying this association within tertiles of BMI, it was found that only in the 2^nd ^tertile the relationship between birth weight and FFM was significant (data not shown). Another significant association was found between birth weight and for SBP, which is in line with others and previously found in the AGAHLS[39,40]. No other significant associations were found, although the associations between birth weight and adult LDL, FM and waist circumference were in the expected (negative) directions [41-46] No associations were found between birth weight and resting heart rate or carotid IMT. This latter finding is in contrast with what has been reported by Leeson et al. [47]. However, their study focussed on an older population.

Birth weight is considered to be mainly dependent on the intra-uterine environment, such as the availability of nutrients and oxygen [30]. In the present population, the *IGF-I *genotype could only explain 6% of the variance in birth weight in men, and only 1% of the variance in birth weight in women. The magnitude of the relationship between birth weight and risk factors for CVD and T2DM, however, seems to be dependent on genes (i.e. *IGF-I *promoter polymorphism) (Table 4). This modification was strongest in the association between birth weight and blood pressure. In the VC group, a 1 kg lower birth weight was found to be related to an 8 mmHg increase in SBP, which is much more than has been reported in the literature (2 to 3 mm Hg) [39]. The relationship between birth weight and DBP was also stronger than was expected (as 1 kg lower birth weight was related to a 5 mmHg increase in DBP). No other study has reported on interactions between genes and birth weight in the relationship with adult blood pressure, although IJzerman et al, in twins studies, have shown that the association between birth weight and blood pressure depends on genetic factors [48,49] Interactions between birth weight and other genes have been reported before, which might suggest that some genotypes are more prone to adverse circumstances during fetal growth, under the assumption that birth weight is mainly dependent on the intra-uterine environment [15-17] On the other hand, the observed interactions between birth weight and *IGF-I promoter *polymorphism genotype could be a result of a gene-gene interaction, since birth weight is also determined by genes (other than the *IGF-I gene*).

This study was conducted in 264 subjects only, which is considered few in studies on genetic associations. This might be a reason that we did not find significant associations between genotype and the risk factors. Subjects born pre term were excluded, since gestational age may be another factor associated with risk factors for CVD and T2DM, but with another underlying mechanism [21]. We reanalysed the data including subjects born pre term but fulfilling the other inclusion criteria (N = 22), which showed some different β 's (birth weight was now significantly associated with FM, waist circumference (unadjusted model) and with DBP pressure (adjusted model)). However, the interaction between birth weight and *IGF-I genotype *was the same, with strong associations between birth weight and SBP and DBP in the VC (β = - 8.40 and β = -5.67, respectively). Furthermore, despite the fact that the AGAHLS is a longitudinal study, no data was available on infant growth, nor reliable data on birth length was available. If so, it was possible to study effects of *IGF-I genotype *on infant growth or interactions with infant growth.

## Conclusion

From this study, it is concluded that *IGF-I promoter *polymorphism genotype is related to birth weight in men only, that this genotype is not associated with risk factors for CVD and T2DM, and, most interestingly, that the *IGF-I promoter *polymorphism genotype modifies the relationship between birth weight and risk factors for CVD and T2DM, especially for SBP and DBP.

## List of abbreviations

β – regression coefficient

95%CI – 95% confidence interval

AGAHLS – Amsterdam Growth and Health Longitudinal Study

BMI – body mass index

bp – base pair

CA – cytosine adenine

CVD – cardiovascular diseases

DBP – diastolic blood pressure

T2DM – type 2 diabetes

FFM – fat-free mass

FM – fat-mass

HbA_1c _– glycated hemoglobin

IGF-1 – insulin like growth factor 1

IMT – intima-media thickness

LDL – low density lipoprotein

SBP – systolic blood pressure

VC – variant carrier

WHR – waist-to-hip ratio

WT – wild type

## Competing interests

The author(s) declare that they have no competing interests.

## Authors' contributions

The first three authors (StV, EvR and PV) equally contributed to the paper. They were involved in the statistical analysis, interpretations of the data, laboratory work to determine the *IGF-I polymorphism *and writing of the paper.

JT, HD, CS, WvM, SL and HK were supervisors and involved in developing the design, assist in statistical analysis and interpretation of the results. They all have been involved in drafting the article or revising it critically for important intellectual content. All authors read and approved the final manuscript.

## Pre-publication history

The pre-publication history for this paper can be accessed here:


